# Tumor-Stroma Proportion to Predict Chemoresistance in Patients With Ovarian Cancer

**DOI:** 10.1001/jamanetworkopen.2024.0407

**Published:** 2024-02-27

**Authors:** Emil Lou, Valentino Clemente, Marcel Grube, Axel Svedbom, Andrew C. Nelson, Freya Blome, Annette Staebler, Stefan Kommoss, Martina Bazzaro

**Affiliations:** 1 Division of Hematology, Oncology, and Transplantation, Department of Medicine, University of Minnesota, Minneapolis; 2 Masonic Cancer Center, University of Minnesota, Minneapolis; 3 Department of Women’s Health, Tübingen University Hospital, Tübingen, Germany; 4 Department of Obstetrics, Gynecology and Women’s Health, University of Minnesota, Minneapolis; 5 Division of Dermatology and Venereology, Department of Medicine, Karolinska Institute, Stockholm, Sweden; 6 Department of Laboratory Medicine and Pathology, University of Minnesota, Minneapolis; 7 Institute of Pathology and Neuropathology, University of Tübingen, Tübingen, Germany

## Abstract

**IMPORTANCE:**

Platinum-based chemotherapy is the backbone of standard-of-care treatment for patients with advanced-stage, high-grade serous carcinoma (HGSC), the most common form of ovarian cancer; however, one-third of patients have or acquire chemoresistance toward platinum-based therapies.

**OBJECTIVE:**

To demonstrate the utility of tumor-stroma proportion (TSP) as a predictive biomarker of chemoresistance of HGSC, progression-free survival (PFS), and overall survival (OS).

**DESIGN, SETTING, AND PARTICIPANTS:**

This prognostic study leveraged tumors from patients with HGSC in The Cancer Genome Atlas (TCGA) cohort (1993-2013) and an independent cohort of resected clinical specimens from patients with HGSC (2004-2014) available in diagnostic and tissue microarray formats from the University of Tübingen in Germany. Data analysis was conducted from January 2021 to January 2024.

**EXPOSURE:**

Diagnosis of HGSC.

**MAIN OUTCOMES AND MEASURES:**

Principal outcome measures were the ability of TSP to predict platinum chemoresistance, PFS, and OS. Using hematoxylin and eosin–stained slides from the Tübingen cohort (used for routine diagnostic assessment from surgical specimens) as well as tissue microarrays, representative sections of tumors for scoring of TSP were identified using previously evaluated cutoffs of 50% stroma or greater (high TSP) and less than 50% stroma (low TSP). Digitized slides from the TCGA Cohort were analyzed and scored in a similar fashion. Kaplan-Meier time-to-event functions were fit to estimate PFS and OS.

**RESULTS:**

The study included 103 patients (mean [SD] age, 61.6 [11.1] years) from the TCGA cohort and 192 patients (mean [SD] age at diagnosis, 63.7 [11.1] years) from the Tübingen cohort. In the TCGA cohort, there was no significant association of TSP levels with chemoresistance, PFS, or OS. However, in the Tübingen cohort, high TSP was associated with significantly shorter PFS (HR, 1.586; 95% CI, 1.093-2.302; *P* = .02) and OS (hazard ratio [HR], 1.867; 1.249-2.789; *P* = .002). Patients with chemoresistant tumors were twice as likely to have high TSP as compared to patients with chemosensitive tumors (HR, 2.861; 95% CI, 1.256-6.515; *P* = .01). In tissue microarrays from 185 patients from the Tübingen cohort, high TSP was again associated with significantly shorter PFS (HR, 1.675; 95% CI, 1.012-2.772 *P* = .04) and OS (HR, 2.491; 95% CI, 1.585-3.912; *P* < .001).

**CONCLUSIONS AND RELEVANCE:**

In this prognostic study, TSP was a consistent and reproducible marker of clinical outcome measures of HGSC, including PFS, OS, and platinum chemoresistance. Accurate and cost-effective predictive biomarkers of platinum chemotherapy resistance are needed to identify patients most likely to benefit from standard treatments, and TSP can easily be implemented and integrated into prospective clinical trial design and adapted to identify patients who are least likely to benefit long-term from conventional platinum-based cytotoxic chemotherapy treatment at the time of initial diagnosis.

## Key Points

**Question:**

Is tumor-stroma proportion (TSP) a predictive biomarker of platinum chemotherapy resistance and prognosis for patients with ovarian cancer?

**Findings:**

This prognostic study of 295 patients with ovarian cancer from 2 distinct cohorts found that a TSP greater than 50% was a predictive biomarker of platinum chemotherapy resistance and was associated with both progression-free survival and overall survival.

**Meaning:**

These findings suggest that TSP should be further standardized and incorporated into prospective clinical trials as a correlative predictive biomarker for drug resistance.

## Teaser

This prognostic study examines the utility of tumor-stroma proportion as a predictive biomarker for chemoresistance among patients with ovarian cancer.

## Introduction

The role of the tumor microenvironment (TME) in predicting invasive behavior, metastatic potential, and chemoresistance of tumors is an emerging field at the intersection of tumor biology and clinical care.^1,2,3,4,5^ Tumor-stroma proportion (TSP) is an assessment of the extent of tumor involvement with stromatous TME components and has been associated with poor prognosis in a broad range of cancers.^6,7,8^ Ovarian cancer is a heterogeneous and potentially invasive form of carcinoma. Nearly 1 out of every 3 patients develops resistance to platinum-based chemotherapy, which remains the backbone of standard-of-care treatment for advanced-stage forms of this disease.^9,10^

New therapeutic targets for the treatment of recurrent ovarian cancer are urgently needed, especially for patients with disease that is refractory to standard-of-care platinum chemotherapy. Even in the era of molecular oncology and advances in our understanding of the genomic factors and passengers associated with the carcinogenesis and metastatic potential of ovarian carcinomas, there is limited efficacy of molecular therapeutics targeting these variants. This state of the field provides an opportunity to examine the biology of the ovarian TME as potential alternative targets for treatment. In 2019, we published results from a prospective study^11^ of patients with newly diagnosed ovarian tumors, which showed that tumors harboring a higher TSP predicted eventual emergence of platinum drug resistance. This study^11^ was, to our knowledge, the first study to evaluate TSP from assessment at time of ovarian cancer diagnosis and provide an association with eventual emergence of drug resistance over time. The patients were monitored over time for response of their tumors to standard-of-care, platinum-based chemotherapy, and their cases were designated as platinum-sensitive or platinum-resistant based on standard definitions in the field (ie, recurrence after 6 months following first-line treatment vs recurrence prior to 6 months following first-line treatment).^11^ TSP assessed from initial biopsy or surgical specimen was determined using slides used for establishing histopathologic diagnosis. The discovery that high TSP was associated with platinum-resistance^11^ offered TSP as a potentially effective and low-cost predictive biomarker of drug resistance that, if confirmed and validated, could advance the field of ovarian cancer treatment by helping to tailor treatment for patients in whom platinum therapy was predicted to be less effective than in others.

Thus, we next sought to confirm these results in a larger patient cohort. In this study, we accessed The Cancer Genome Atlas Program (TCGA) high-grade serous carcinoma (HGSC) cohort and created a larger collaborative network with access to specimens of patients with ovarian carcinoma linked to clinical outcomes data that included information on progression-free survival (PFS), overall survival (OS), and platinum chemotherapy resistance. We excluded intrinsic variability due to different biology of multiple histologic subtypes of ovarian cancer by choosing to use HGSC only. We performed bivariable and multivariable analysis of chemoresistance and survival in this cohort and smaller cohorts in order to exclude the effect of confounders and identify specific subpopulations that may benefit the most from the use of TSP as a predictive biomarker.

## Methods

This prognostic study was approved by the University of Minnesota institutional review board and followed the Transparent Reporting of a Multivariable Prediction Model for Individual Prognosis or Diagnosis (TRIPOD) reporting guideline for reporting prediction model validation. Patients provided written informed consent for use of surgical tissue specimens for research. Archival tissues were used with approval from the internal ethics review board (Ethikkommission) of the Medical Faculty of the University of Tübingen. The study was performed in accordance with the Declaration of Helsinki.^12^

### TCGA Cohort

We interrogated the TCGA dataset for cases of ovarian cancer which had annotated clinical data and hematoxylin and eosin (H&E)–stained slides that had been used for diagnosis and then scanned into the TCGA archives. This search was performed using the following search terms and filters: *ovary *for the primary site filter, *slide image *for the data type filter, *diagnostic slide* for the experimental strategy filter, and *cystic, mucinous, and serous neoplasms* for the disease type filter. This algorithm retrieved a total of 106 cases. Three patients were excluded due to the absence of clinical data or poor quality of the specimen. The final cohort consisted of patients whose cases were diagnosed and treated at multiple institutions between 1993 and 2013. *The International Classification of Diseases for Oncology, Third Edition* (*ICD-O-3*) codes for topographic origin were C56.9 (malignant neoplasm of the ovary) and C48.0 (malignant neoplasm of retroperitoneum). *ICD-O-3* codes for pathological diagnosis were: 8441/3 (serous cystadenocarcinoma, including serous adenocarcinoma and serous carcinoma), 8440/3 (cystadenocarcinoma) and 8460/3 (serous cystadenocarcinoma). All patients were treated with surgery followed by standard-of-care adjuvant chemotherapy. No history of neoadjuvant chemotherapy was reported.

### Tübingen Cohort

The Tübingen cohort consisted of clinical specimens derived from patients assessed at Tübingen University Hospital in Tübingen, Germany from 2004 to 2014 for suspected or known ovarian carcinomas, and who underwent debulking surgical resection of tumors followed by standard-of-care systemic therapy. Demographic information and patient characteristics are reported in eTable 1 in Supplement 1.

### TSP Scoring 

The scoring was performed by 1 clinician (V.C.) and 1 pathologist (A.N.) who examined the H&E–stained slides at ×10 magnification to identify representative sections of the primary tumor. For scoring of the whole sections, after identifying the most densely populated cellular areas of the slide, the relative amounts of tissue represented by cancer cells vs. stromal tissue were identified and labeled using 50% as a previously validated cutoff.^11^ Tumors with a stroma proportion less than 50% were classified as low TSP (TSP = 0) and tumors with a stroma proportion of 50% or greater were classified as high TSP (TSP = 1). Stromal tissue that was not in clear continuity with the peritumoral stroma was not included in the evaluation. Necrotic, mucinous, empty, or large inflammatory areas were excluded from the scoring.

Many studies retrospectively assessing reactive tumor stroma patterns use an approach based on tissue microarrays (TMA) constructed using available resection specimens. Because this approach is commonly used, we wanted to confirm whether the TMA approach would yield the same results as whole-slide assessment; this difference in tumor preparation could account for potential differences in outcomes. These TMAs consisted of 6 punches for each clinical specimen built using 0.8 mm diameter cores (6 for each tumor). A total of 1110 H&E–stained slides were identified and labeled using 50% as the cutoff point. A single TSP category was assigned to each patient based on the mode from the 6 total TSPs values. Based on this process, patients were labeled as stroma-poor (ie, TSP = 0 [low TSP]) or stroma-rich (ie, TSP = 1 [high TSP]). Finally, the univariable of the outcome was performed using standard Kaplan-Meier survival curves and log-rank tests to compare PFS and OS for patients with high TSP and low TSP.

### Covariates

In multivariable analysis we adjusted for age at diagnosis, residual disease, primary metastases (yes or no), and lymph node spread (yes or no). Chemoresistance was defined as progressive disease during chemotherapy or less than 180 days after the first adjuvant regimen. Residual disease was reported as: (1) no residual disease, (2) residual disease 0 (ie, <1 cm remaining following suboptimal debulking), or (3) residual disease 1 (ie, ≥1 cm remaining following optimal debulking). All covariates were measured at baseline.

### Statistical Analysis 

The analyses were performed using Stata statistical software version 17.2 (StataCorp); graphs and analyses shown were performed using GraphPad Prism version 8.4.3 (Domatics). As for these analyses, Kaplan-Meier curves and the associated Mantel-Haenszel hazard ratios (HRs) and *P* values (log rank test) were generated using the standard survival function for comparing 2 groups in GraphPad Prism. We presented categorical variables using frequency (percentage), and continuous variables using mean (SD) or median (IQR) depending on the approximate normality of the underlying distributions. We compared categorical variables using χ^2^ tests and continuous variables using *t* tests by virtue of the central limit theorem. In time-to-event analyses, patients were followed until first of failure or right-censoring, defined as loss to follow-up or date of data extraction. Kaplan-Meier time-to-event functions^13^ were fit to estimate PFS and OS and we fit Cox proportional hazards models^14^ to address confounding factors. We tested for differences in time-to-events between groups using the Wald statistic for the HR from Cox proportional hazards models and validated the proportionality assumption by inspecting the Schoenfeld residuals.^15^ The proportionality assumption was valid unless otherwise noted. We fit a logistic regression model to estimate the association of platinum resistance with TSP, controlling for the covariates (age at diagnosis, lymph node spread [yes or no], distant metastasis [yes or no], and residual disease [yes or no]). A 2-sided P < .05 was considered statistically significant. Data analysis occurred from January 2021 to January 2024.

## Results

### Patient Demographics and TSP Assessment in the TCGA Cohort

**Figure 1.**
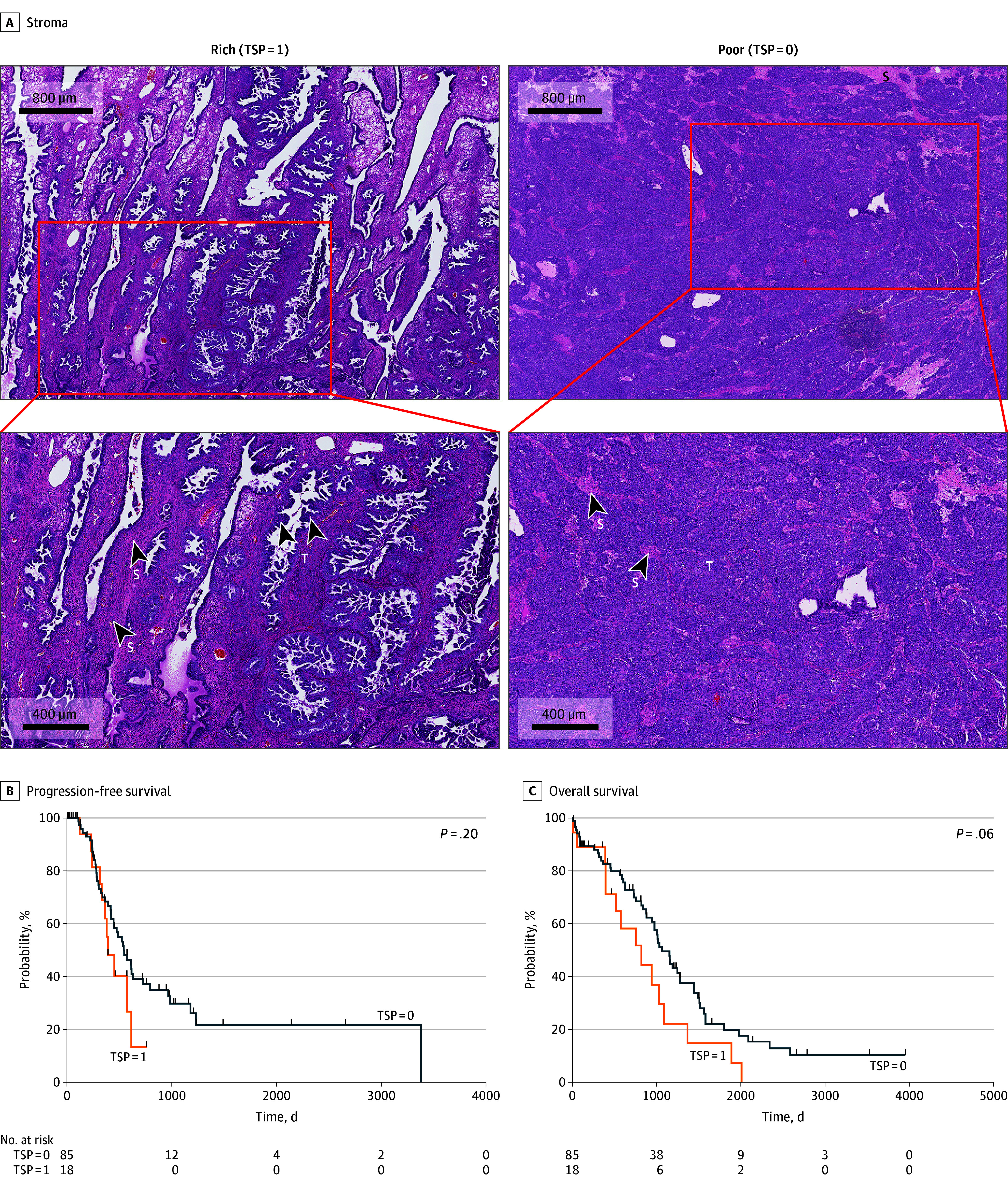
Association of Tumor-Stroma Proportion (TSP) and Clinical Outcomes in Patients With Ovarian Cancer in the The Cancer Genome Atlas (TCGA) Cohort The figure shows representative images (A) of stroma-rich (high TSP [TSP = 1]) and stroma-poor (low TSP [TSP = 0]) specimens in the TCGA cohort. S indicates the stromal component within the tumor; T, cancerous cells within the specimen. The Kaplan-Meier curves show progression-free survival (B) in patients with low TSP vs high TSP (HR, 1.661; 95% CI, 0.766-3.603) and overall survival (C) in patients with low TSP vs high TSP (HR 1.906; 95% CI, 0.962-3.776 95%).

The TCGA cohort consisted of 103 patients (mean [SD] age, 61.6 [11.1] years) with a histopathologic diagnosis of ovarian carcinomas. Patient demographics and clinical characteristics, including International Federation of Gynecology and Obstetrics (FIGO) stage, residual disease following surgery, and chemoresistance are listed in Table 1. Most patients (73 patients [70.9%]) were FIGO stage III, and 76 patients (73.8%) had residual disease following surgical debulking. Although only 12 patients (11.7%) had chemoresistant disease, 31 patients (30.1%) had unknown chemoresistance status, which limited the ability to provide confirmation in this analysis. Representative images of H&E–stained slides of TSP = 0 and TSP = 1 are shown in Figure 1A.

**Table 1.** Patient Demographics, Clinical Characteristics, and TSP of the Patients Diagnosed with Ovarian Carcinoma in The Cancer Genome Atlas Cohort

Characteristic	Patients, No. (%)^a^			*P* value^b^
	All (N = 103)	TSP = 0 (n = 85)	TSP = 1 (n = 18)	
Age at diagnosis, mean (SD), y	61.6 (11.1)	61.4 (11.2)	62.7 (11.1)	.66
International Federation of Gynecology and Obstetrics stage				.91
Stage IC	2 (1.9)	2 (2.3)	0	
Stage IIA	1 (1.0)	1 (1.2)	0	
Stage IIB	1 (1.0)	1 (1.2)	0	
Stage IIC	2 (1.9)	1 (1.2)	1 (5.6)	
Stage IIIB	1 (1.0)	1 (1.2)	0	
Stage IIIC	72 (69.9)	59 (69.4)	13 (72.2)	
Stage IV	23 (22.3)	19 (22.3)	4 (22.2)	
Unknown	1 (1.0)	1 (1.0)	0	
Residual disease				.76
No	21 (20.4)	18 (21.1)	3 (16.6)	
Yes	76 (73.8)	63 (74.1)	13 (72.2)	
Unknown	6 (5.8)	4 (4.7)	2 (11.1)	
Chemoresistance				.78
No	60 (58.2)	49 (57.6)	12 (66.7)	
Yes	12 (11.7)	12 (14.1)	2 (11.1)	
Unknown	31 (30.1)	24 (28.2)	4 (22.2)	

Abbreviation: TSP, tumor-stroma proportion.

^a^ Cases with low TSP (<50%) are labeled as TSP = 0 and cases with high TSP (≥50%) are labeled as TSP = 1.

^b^ Comparisons of categorical variables were conducted using Pearson χ^2^ tests, and the comparison of age between the groups was conducted using a *t* test with pooled variance.

### Characteristics of Patients With High vs Low TSP in the TCGA Cohort

We compared patient demographic characteristics, including age at diagnosis, stage, presence of residual disease, and platinum-resistant vs platinum–sensitive forms of the disease with TSP (Table 1). Of the 103 patients in the TCGA data set, 18 patients (17.5%) had high TSP (TSP = 1) and the remaining 85 patients had low TSP (TSP = 0). No significant differences were found between patients with high or low TSP when comparing clinical and demographic characteristics.

### PFS and OS in the TCGA Cohort

We compared outcomes by TSP status including PFS and OS in the TCGA cohort. Our analysis revealed that a TSP = 1 status was not associated with worse PFS several following initial diagnosis and treatment (HR, 1.661; 95% CI, 0.7661-3.603; *P* = .20) (Figure 1B). Additionally, a status of TSP = 1 was not associated with OS (HR, 1.906; 95% CI, 0.9622-3.776; *P* = .06), again following a similar pattern that differentiated the 2 groups after the first several years from diagnosis. (Figure 1C). We observed no significant association of TSP levels with chemoresistance in the TCGA cohort (Table 1); as noted above, chemoresistance status was unknown or otherwise not listed for 31 patients in this data set. Thus, overall, further investigation would be needed using a larger and more complete data set to determine if there is an association of high TSP with worse PFS.

### Patient Demographics and TSP Assessment in the Tübingen Cohort

**Figure 2.**
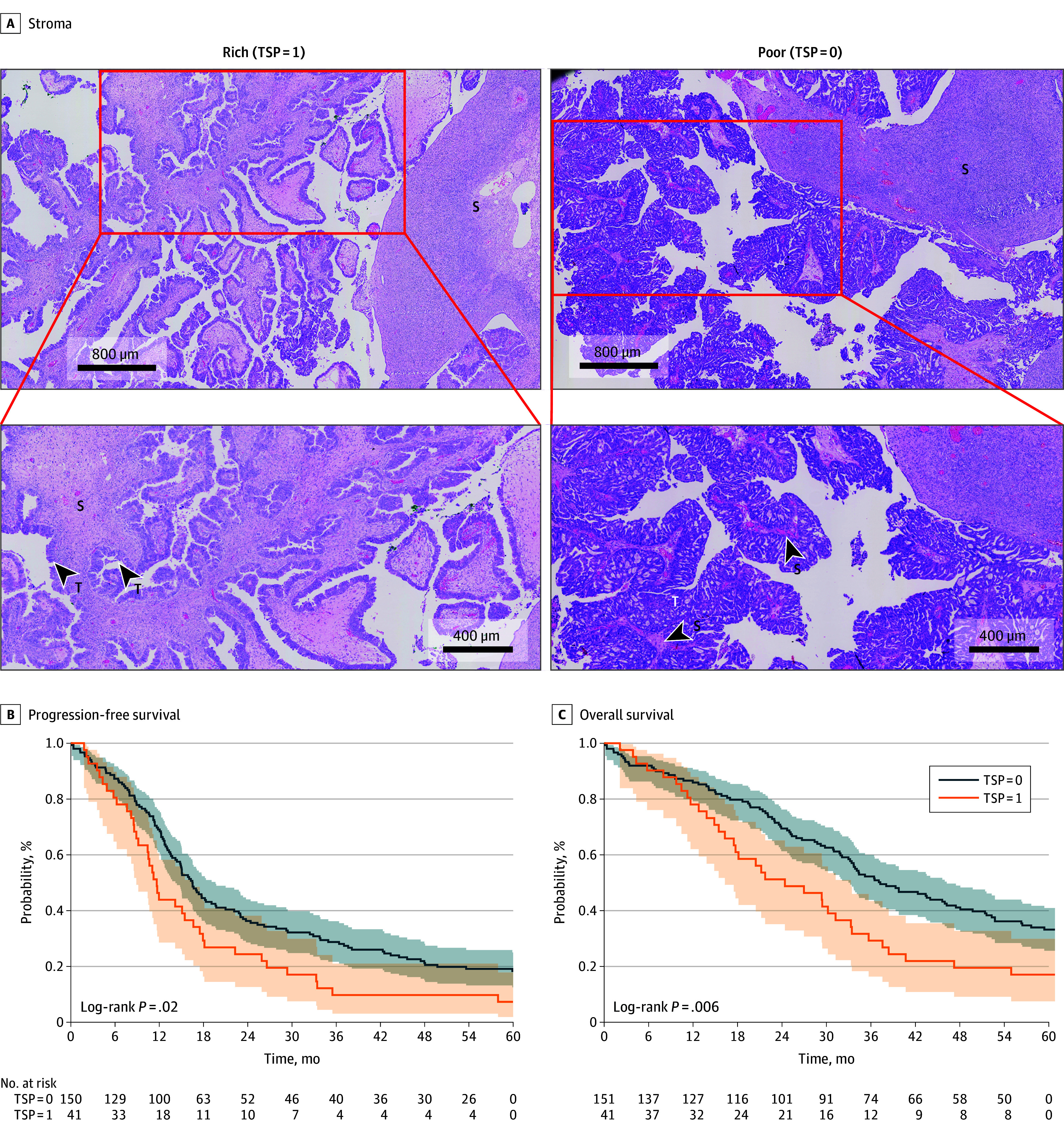
Association of Tumor-Stroma Proportion (TSP) and Clinical Outcomes in Patients With Ovarian Cancer in the Tübingen Cohort The figure shows representative images (A) of stroma-rich (high TSP [TSP = 1]) specimens and stroma-poor (low TSP [TSP = 0]) specimens in the Tübingen cohort. S indicates the stromal component within the tumor; T, cancerous cells within the specimen. The Kaplan-Meier curves show progression-free survival (B) in patients with low TSP vs high TSP (HR, 1.573; 95% CI, 1.090-2.270; *P* = .02) and overall survival (C) in patients with low TSP vs high TSP (HR, 1.726; 95% CI, 1.166-2.557; *P* = .006). The shading represents the 95% CIs.

Having previously demonstrated a significant association of TSP with ovarian cancer chemoresistance in a small prospective cohort,^11^ but no statistically significant association using the larger but limited TCGA data set, we next sought confirmation using the Tübingen cohort, which was larger and more comprehensively annotated. We performed a retrospective analysis of the 192 patients in this cohort (mean [SD] age at diagnosis, 63.7 [11.1] years) who were diagnosed with HGSC of the ovaries. Patient demographics and clinical characteristics, including FIGO stage and presence of residual disease following surgery, are listed in eTable 1 in Supplement 1. The majority of patients (134 patients [69.8%]) were FIGO stage III, and 95 patients (49.5%) had node-positive disease. A total of 132 patients (68.8%) had no evidence of distant metastasis. Over one-half of patients (108 patients [56.3%]) had residual disease following surgical debulking. Representative images of H&E–stained slides from the Tübingen cohort of patients with high TSP and low TSP are provided in Figure 2A.

### Characteristics of Patients With High vs Low TSP in the Tübingen Cohort

We compared patient demographic characteristics including age at diagnosis, stage, presence of residual disease, and platinum-resistant vs platinum-sensitive forms of disease with TSP (Table 2). Of the total 192 patients in the data set, 41 patients (21.4%) had tumors with high TSP, and the remaining 151 patients (78.6%) had tumors with low TSP. Patients with high TSP had a higher rate of FIGO stage IV cases at time of initial diagnosis (14 of 41 patients [34.1%] compared with patients with low TSP (22 of 151 patients [14.6%]; *P* = .04). The most frequent T and N stages in both groups were T3C and N0, respectively; this is likely explained by the higher rate of distant metastases in patients with high TSP (14 of 41 patients [34.1%]) vs in patients with low TSP (22 of 151 patients [14.6%]; *P* = .01). There were no other significant differences in age, tumor size, number, and extent of affected lymph nodes between patients with high vs low TSP.

**Table 2.** Patient Demographics and Clinical Characteristics of Patients With High Grade Serous Carcinoma in Divided by TSP in the Tübingen Cohort

Characteristic	Patients, No. (%) (N = 192)^a^		*P* value^b^
	TSP = 0 (n =151)	TSP = 1 (n =41)	
Age at diagnosis, mean (SD), y	63.6 (10.9)	64.2 (11.8)	.74
International Federation of Gynecology and Obstetrics stage			
1	6 (4.0)	2 (4.9)	.04
2	12 (7.9)	2 (4.9)	
3	111 (73.5)	23 (56.1)	
4	22 (14.6)	14 (34.1)	
Tumor category			
T1	7 (4.6)	2 (4.9)	.50
T2	40 (26.5)	7 (17.1)	
T3	100 (66.2)	30 (73.2)	
Unknown	4 (2.6)	2 (4.9)	
Lymph node category			
N0	58 (38.4)	17 (41.5)	.65
N1	77 (51.0)	18 (43.9)	
Unknown	16 (10.6)	6 (14.6)	
Metastases			
M0	107 (70.9)	25 (61.0)	.01
M1	22 (14.6)	14 (34.1)	
Unknown	22 (14.6)	2 (4.9)	
Residual disease			
No	61 (40.4)	19 (46.3)	.58
Yes	86 (57.0)	22 (53.7)	
Unknown	4 (2.6)	0	
Platinum resistance			
No	129 (85.4)	27 (65.9)	.004
Yes	22 (14.6)	14 (34.1)	

Abbreviation: TSP, tumor-stroma proportion.

^a^ Cases with low TSP (<50%) are labeled as TSP = 0 and cases with high TSP (≥50%) are labeled as TSP = 1.

^b^ Comparisons of categorical variables were conducted using Pearson χ^2^ tests, and the comparison of age between the groups was conducted using a *t* test with pooled variance.

Approximately 25% to 30% of patients with HGSC develop platinum-resistant tumors.^9,10^ We have previously reported that high TSP is predictive of platinum resistance^11^; thus, in this cohort, we compared patients with TSP = 0 vs TSP = 1 and their response to platinum-based chemotherapy. There was a significantly higher proportion of TSP = 1 cases that were platinum-resistant (14 of 41 cases [34.1%]) as compared with TSP = 0 cases (22 of 151 cases [14.6%]; (*P* = .004) (Table 2). In contrast, more TSP = 0 cases were platinum-sensitive (129 of 151 cases [85.4%]) than was seen in TSP = 1 cases (27 of 41 cases [65.9%]).

### PFS and OS in All Patients With HGSC in the Tübingen Cohort

Among all patients with HGSC, patients with low TSP had a higher probability of PFS than patients with high TSP at all-time points during follow-up (HR, 1.573; 95% CI, 1.090-2.270; *P* = .02) (Figure 2B). This was also true when we adjusted for age at diagnosis, primary metastasis, and residual disease, at which time TSP = 1 was significantly associated with lower PFS (HR, 1.586; 95% CI, 1.093-2.302; *P* = .02) (eTable 2 in Supplement 1). The presence of residual disease after surgery—an established prognostic factor for ovarian cancer—was also associated with worse PFS (HR, 2.038; 95% CI, 1.436-2.892; *P* < .001). However, the presence of distant metastases, which was more common in the TSP = 1 group, was not associated with PFS following adjustment for contributions of other variables.

In terms of OS, patients with TSP = 0 also had a higher OS at all time points except for the initial months (HR, 1.726; 95% CI, 1.166-2.557; *P* = .006) (Figure 2C). When we adjusted for age at diagnosis, primary metastasis, and residual disease, TSP = 1 was significantly associated with lower OS (HR, 1.867; 95% CI, 1.249-2.789; *P* = .002) (eTable 3 in Supplement 1). As seen with PFS, the presence of residual disease was associated with shorter OS (HR, 2.604; 95% CI, 1.764-3.844; *P* < .001), as were metastases (HR, 1.572; 95% CI, 1.040-2.375; P = .03) and age (HR, 1.035; 95% CI, 1.018-1.052; *P* < .001).

### TMA

Of the 192 clinical specimens in the Tübingen cohort, 185 were available in the TMA format for this next analysis. Demographic characteristics are presented in eTable 1 in Supplement 1. Representative images of the TMA sections are shown in eFigure 1 in Supplement 1. Of these 185 TMA-constructs, we designated 143 as low TSP (stroma-poor), and 42 as high TSP (stroma-rich). These numbers presented a 15.6% discrepancy between the whole-slide, H&E–stained diagnostic slides presented above and the TMA-based scoring (eFigure 2 in Supplement 1). Nonetheless, univariable analysis of these TMA-constructs again confirmed a statistically significant association of high TSP with lower PFS (HR, 1.675; 95% CI, 1.012-2.772; *P* = .04) and OS (HR, 2.491; 95% CI, 1.585-3.912; *P* < .001) in the Tübingen cohort (eFigure 1 in Supplement 1).

### Confirmation of the Association of TSP With Platinum Chemoresistance in Patients With HGSC in the Tübingen Cohort

**Figure 3.**
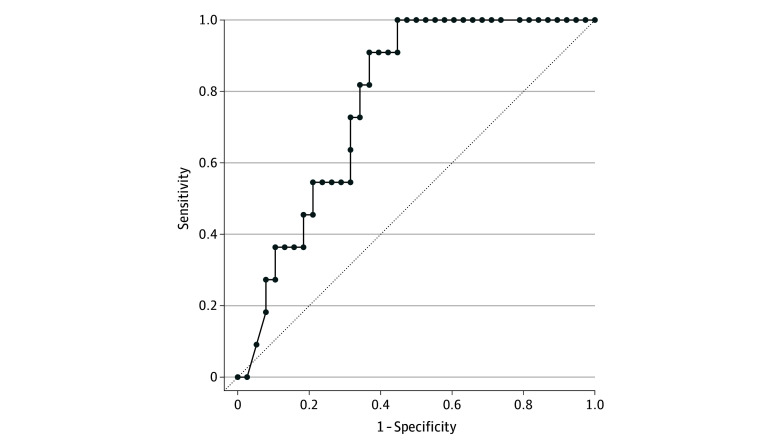
Stroma-Rich Tumors as Predictors of Platinum Resistance in the Tübingen Cohort Receiver operating characteristic (ROC) curve of stroma-rich tumors (TSP = 1) and risk factors (metastasis, residual disease, and lymph node spread) indicating the ability of TSP = 1 to differentiate chemoresistant and chemosensitive patients. The diagonal line indicates no predictive value. The area under the ROC curve is 0.7644 (95% CI, 0.639-0.889).

Finally, we used the Tübingen cohort to validate our previously reported findings from 2019^11^ that high TSP is indeed associated with emerging platinum chemotherapy resistance in patients with ovarian cancer. We performed multivariable analysis of cases based on chemoresistance and confirmed a statistically significant difference in which high TSP was associated with platinum-chemoresistance (odds ratio, 2.861; 95% CI, 1.256-6.515; *P* = .01) after adjusting for age, primary metastasis, and residual disease (eTable 4 in Supplement 1). Furthermore, the area under the receiver operating characteristic curve for the model predicting platinum resistance based on age at diagnosis, metastasis, residual disease, lymph node spread, and TSP was estimated at 0.7644 (95% CI, 0.639-0.889) (Figure 3). Taken together, these results not only suggest that chemoresistance in patients with TSP = 1 is associated with poorer outcomes also observed in these patients, but also suggest that TSP may have specificity and sensitivity high enough to be used in the clinical setting to predict which patients are going to respond to treatments or benefit from newer therapeutic options.

## Discussion

TSP is a readily assessable property of the TME that holds strong promise not just as an indicator of prognosis, but perhaps more importantly as a predictive biomarker of likely emergence of resistance to standard-of-care chemotherapy over time. TSP has been published widely and defined in previous studies^7,8^ in a binary fashion; in other words, as tumors with the stromatous portion comprising less than or more than 50% stroma. In 2019, we reported our finding from a small prospective cohort of patients that TSP is associated with platinum chemoresistance.^11^ In this prognostic study, we present further supportive evidence that high TSP is associated with platinum chemoresistance and also lower PFS and OS in patients with ovarian cancer, using a confirmatory data set focused on 192 patients with a histopathologic diagnosis of HGSC.

In the current study, high TSP was the only factor significantly predictive of emergence of platinum chemotherapy resistance (*P* = .01) following first-line treatment. The risk was even higher in patients with no metastases at diagnosis. These results not only confirm that higher TSP is associated with development of chemoresistance to current standard-of-care treatments in a large cohort of patients with HGSC, but also potentially support the notion that high TSP might be the underlying mechanism of the poorer outcome also observed in these patients. Further studies would be needed to determine factors associated with TSP and chemoresistance at the cellular and molecular level. Our team has investigated molecular and cellular factors that induce and propagate emergence of chemoresistance in patients with ovarian cancer,^13,14,15,16^ including tumor cell communication networks that bridge malignant cells distributed throughout heterogeneous stroma-rich tumors that present with higher TSP.^17^ These communication networks are induced by hypoxia^18^ and other characteristic properties of proliferating tumors^19^ and are just 1 example of factors underlying with association of TSP with ovarian cancer chemoresistance.

The data presented here provide confirmation of high TSP as a useful, companion-predictive biomarker for future emergence of platinum resistance of ovarian carcinomas, and also support the notion that it can predict worse prognosis as well. At face value, these results differ from recently reported results from Micke et al,^6^ who examined prognostic impact of tumor-stroma fraction in 2732 cases of resected tumors representing 16 separate solid tumor types; of this total, there were 197 cases categorized as ovarian carcinoma, with an additional 49 cases listed as HGSC. The investigators used TMA-constructs, which they acknowledged carry limitations in potential biasing of assessable portions of resected tumors in context of intratumoral heterogeneity.^6^ Using deep learning methods, they identified and quantified stroma and analyzed differences using median levels of stroma fraction as the primary cutoff. The median cutoff used for differentiating stroma-rich and stroma-poor fractions was well below 50% for ovarian cancer cases overall (150 cases reported) and high-grade serous ovarian cancer (49 cases).^6^ The authors determined that there was no significant association of TSP with prognosis in many cancer types tested in their study, including both categories of ovarian carcinomas, within the context of reliance on TMAs and the variable cutoff between different tumor types.^6^ For our study, because we sought to confirm our prior findings on chemoresistance,^11^ and to be consistent with other studies that used a 50% cutoff in ovarian and other cancers,^7,20,21,22^ we opted to use this 50% value. We also analyzed both TMA and whole-slide sections of surgical specimens to confirm consistency of findings between both preparative techniques, and, indeed, we confirmed the findings as described.

We found that TSP was also associated with metastases at diagnosis. The M1 cases in the data set included both FIGO stage IVA (ie, intraperitoneal metastases) and FIGO Stage IVB (ie, extraperitoneal metastases), were provided together in our clinical data set, and were unassociated with the site of metastatic disease. It is conceivable that there are differences in platinum resistance between FIGO stage IVA and IVB cases which were not uncovered in this study.

Overall, these data support the notion that higher TSP is associated with HGSC and may be an underlying mechanism in more aggressive forms of HGSC. These results serve as confirmation of our findings in a smaller prospective cohort^11^ from which we first reported the potential predictive association of TSP with chemoresistance in ovarian cancer. This finding suggests that high TSP could help identify patients who may benefit more from alternatives to platinum-based regimens; this would be particularly useful for patients with the most common stage at diagnosis in HGSC and would help determine who could achieve the best therapeutic results based on chemotherapy.

Here, we provide confirmation and validation of our previous finding^11^ associating high TSP with chemoresistance using this larger data set that high TSP is not only predictive of chemotherapy resistance, but also associated with worse PFS and OS, and thus worsened prognosis, in patients with HGSC. While our results align with previous studies^23,24^ suggesting that the stromal gene cluster is associated with unfavorable PFS and OS outcomes in patients with ovarian cancer, our findings further indicate that this association is primarily related to the quantity or extent of stromal involvement.

### Limitations

Limitations of this study include limitations inherent in any retrospective data set, the relatively inexact nature of assessment of residual disease following attempt at maximal cytoreduction and debulking, and the manual process required to review slides for TSP assessment. In general, some of the subset sample sizes were small when analyses were stratified by metastasis and then further split into exploration and validation data sets. This resulted, for instance, in the TCGA cohort not being sufficiently large to find statistically significant differences between TSP = 1 and TSP = 0 ovarian cancers in terms of both demographic characteristics and survival. In addition, intratumoral heterogeneity is a known entity that is an inherent factor in any tissue-based evaluation study; whether TSP varies widely enough within each ovarian tumor is a point of speculation and an unknown factor. Furthermore, intertumoral heterogeneity of TSP between primary ovarian tumors and metastatic tumors to distant sites, which may be enriched in mesenchymal subpopulations and may have inherently high TSP, remains an additional aspect that can be assessed in future prospective validation studies.

## Conclusions

In this prognostic study, findings suggest that high TSP can serve a predictive biomarker of platinum chemotherapy resistance in ovarian carcinomas. This marker is particularly useful for evaluating prognosis as well as drug resistance in cases of HGSOC that undergo maximal debulking surgery, which is usually associated with the best possible prognosis. Here, we demonstrate that TSP serves as an additional stratifying factor for determining cases at highest risk of recurrence and death that are likely due in part to lack of efficacy of standard platinum-based chemotherapies. With this confirmation, we propose that TSP should be further standardized and incorporated into prospective clinical trials as a correlative predictive biomarker for drug resistance.

## Notes

Supplement 1. **eTable 1.** Patient Demographics and Clinical Characteristics of 192 Women Diagnosed with High Grade Serous Carcinoma (HGSC) of the Ovaries (University of Tübingen Cohort) **eTable 2.** Multivariate Analysis of Progression Free Survival **eTable 3.** Multivariate Analysis of Overall Survival **eFigure 1.** Validation of the Use of TMAs to Predict Overall and Progression-Free Survival Based on TSP in the Tübingen Cohort **eFigure 2.** Comparison of TSP Assessment in TMAs With Its Assessment in Slides **eTable 4.** Multivariate Analysis Chemoresistance

Supplement 2.
Data Sharing Statement
