# Supplementary material for: Tumor-Stroma Proportion to Predict Chemoresistance in Patients With Ovarian Cancer
Source: JAMA Netw Open. 2024 Feb 27;7(2):e240407. doi: 10.1001/jamanetworkopen.2024.0407 (PMC10900967; doi:10.1001/jamanetworkopen.2024.0407)
Supplement: Supplement 1. — eTable 1. Patient Demographics and Clinical Characteristics of 192 Women Diagnosed with High Grade Serous Carcinoma (HGSC) of the Ovaries (University of Tübingen Cohort) eTable 2. Multivariate Analysis of Progression Free Survival eTable 3. Multivariate Analysis of Overall Survival eFigure 1. Validation of the Use of TMAs to Predict Overall and Progression-Free Survival Based on TSP in the Tübingen Cohort eFigure 2. Comparison of TSP Assessment in TMAs With Its Assessment in Slides eTable 4. Multivariate Analysis Chemoresistance [file jamanetwopen-e240407-s001.pdf]

Supplemental Online Content

Lou E, Clemente V, Grube M, et al. Tumor-stroma proportion to predict chemoresistance in patients with ovarian cancer. *JAMA Netw Open*. 2024;7(2):e240407. doi:10.1001/jamanetworkopen.2024.0407

eTable 1. Patient Demographics and Clinical Characteristics of 192 Women Diagnosed with High Grade Serous Carcinoma (HGSC) of the Ovaries (University of Tübingen Cohort)
eTable 2. Multivariate Analysis of Progression Free Survival
eTable 3. Multivariate Analysis of Overall Survival
eFigure 1. Validation of the Use of TMAs to Predict Overall and Progression-Free Survival Based on TSP in the Tübingen Cohort
eFigure 2. Comparison of TSP Assessment in TMAs With Its Assessment in Slides
eTable 4. Multivariate Analysis Chemoresistance

This supplemental material has been provided by the authors to give readers additional information about their work.

© 2024 Lou E, et al. *JAMA Network Open.*

eTable 1. Patient Demographics and Clinical Characteristics of 192 Women Diagnosed with High Grade Serous Carcinoma (HGSC) of the Ovaries (University of Tübingen Cohort). SD=standard deviation; FIGO=Federation Internationale de Gynecologie et d'Obstetrique.; TMA=Tissue MicroArray

Characteristic	Level	Slides	TMA
**Number of patients**		192	185
**Age at diagnosis, mean (SD)**		63.7 (11.1) (n=192)	63.4 (11.4)
**Histology**	HGSC	192 (100.0%)	185 (100%)
**FIGO Stage**	1	8 (4.2%)	6 (3.2%)
	2	14 (7.3%)	14 (7.6%)
	3	134 (69.8%)	129 (69.7%)
	4	36 (18.8%)	36 (19.5%)
**Tumor (T)**	1	9 (4.7%)	7 (3.8%)
	2	47 (24.5%)	45 (24.3%)
	3	130 (67.7%)	127 (68.6%)
	Unknown	6 (3.1%)	6 (3.2%)
**Nodes (N)**	N0	75 (39.1%)	70 (37.8%)
	N1	95 (49.5%)	94 (50.8%)
	Unknown	22 (11.5%)	21 (11.4%)
**Metastases (M)**	0	132 (68.8%)	126 (68.1%)
	1	36 (18.8%)	36 (19.5%)
	Unknown	24 (12.5%)	23 (12.4%)
**Residual Disease**	No	80 (41.7%)	74 (40%)
	Yes	108 (56.3%)	107 (57.8%)
	Unknown	4 (2.1%)	4 (2.2%)

eTable 2. Multivariate Analysis of Progression Free Survival

Variable	Haz. ratio	P-value	[95% conf.	interval]
**TSP**	1.586	**0.02**	1.093	2.302
**Age**	1.010	0.19	0.995	1.024
**Metastasis**	1.138	0.53	0.761	1.703
**Residual Disease**	2.038	<0.001	1.436	2.892

eTable 3. Multivariate Analysis of Overall Survival

Variable	Haz. ratio	P-value	[95% conf.	interval]
**TSP**	1.867	**0.002**	1.249	2.789
**Age**	1.035	<0.001	1.018	1.052
**Metastasis**	1.572	0.03	1.040	2.375
**Residual Disease**	2.604	<0.001	1.764	3.844

© 2024 Lou E, et al. *JAMA Network Open.*

eFigure 1. Validation of the Use of TMAs to Predict Overall and Progression-Free Survival Based on TSP in the Tübingen Cohort.

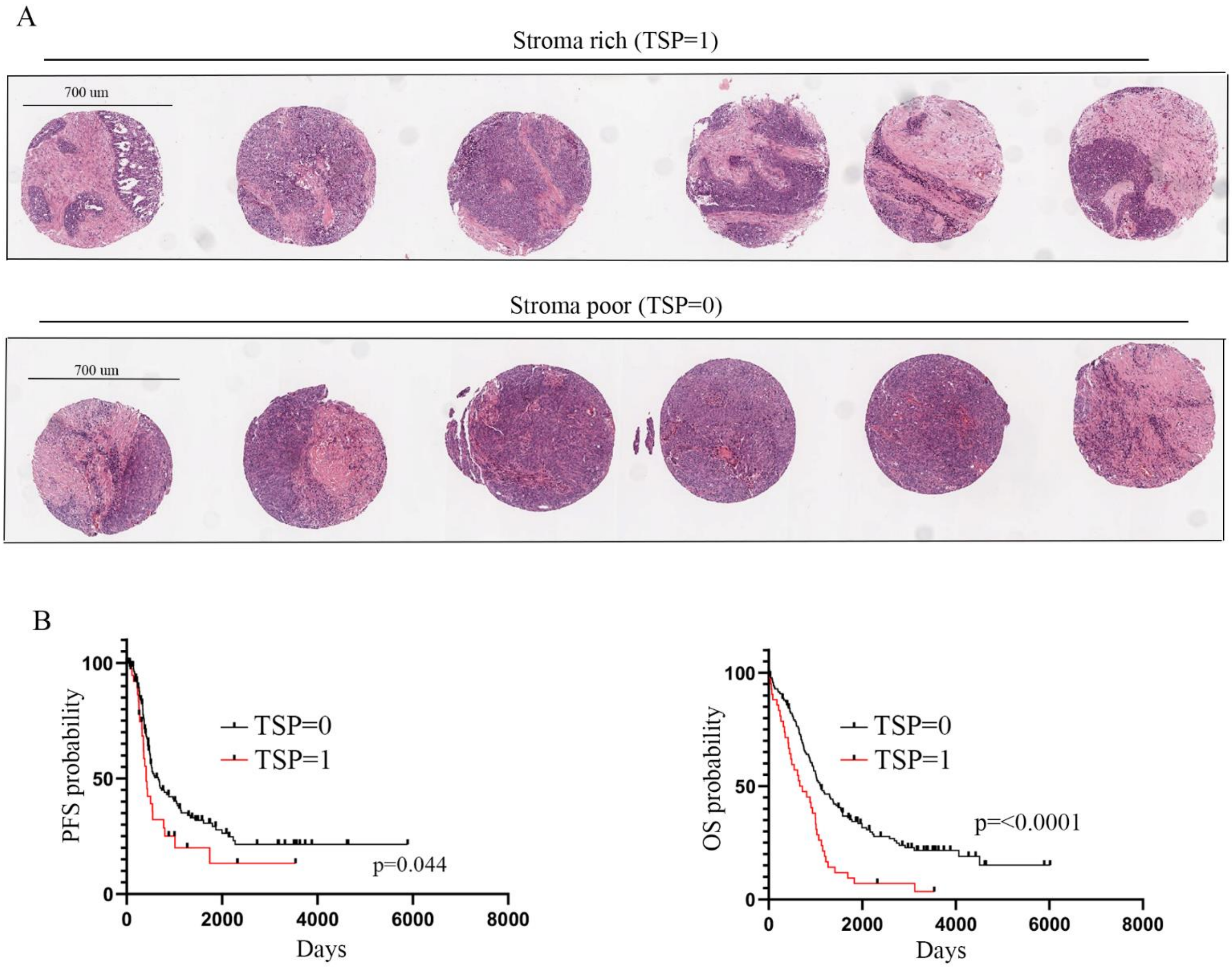

Hazard Ratio (Mantel-Haenszel)	A/B	B/A
Ratio (and its reciprocal)	0.5972	1.675
95% CI of ratio	0.3608 to 0.9884	1.012 to 2.772

Hazard Ratio (Mantel-Haenszel)	A/B	B/A
Ratio (and its reciprocal)	0.4015	2.491
95% CI of ratio	0.2556 to 0.6307	1.585 to 3.912

A. *Top,* representative images of stroma rich. *Bottom,* representative images of stroma poor. **B.** *Left,* Kaplan-Meier curve for progression-free survival (PFS) in patients with low TSP (TSP=0) vs. high TSP (TSP=1) (p=0.04; HR 1.675 95% CI 1.012-2.772) as defined by scoring TMAs. *Right,* Kaplan-Meier curve for overall survival (OS) in patients with low TSP (TSP=0) vs. high TSP (TSP=1) as defined by scoring TMAs (p<0.001; HR 2.491 95% CI 1.585-3.912)

© 2024 Lou E, et al. *JAMA Network Open.*

eFigure 2. Comparison of TSP Assessment in TMAs With Its Assessment in Slides

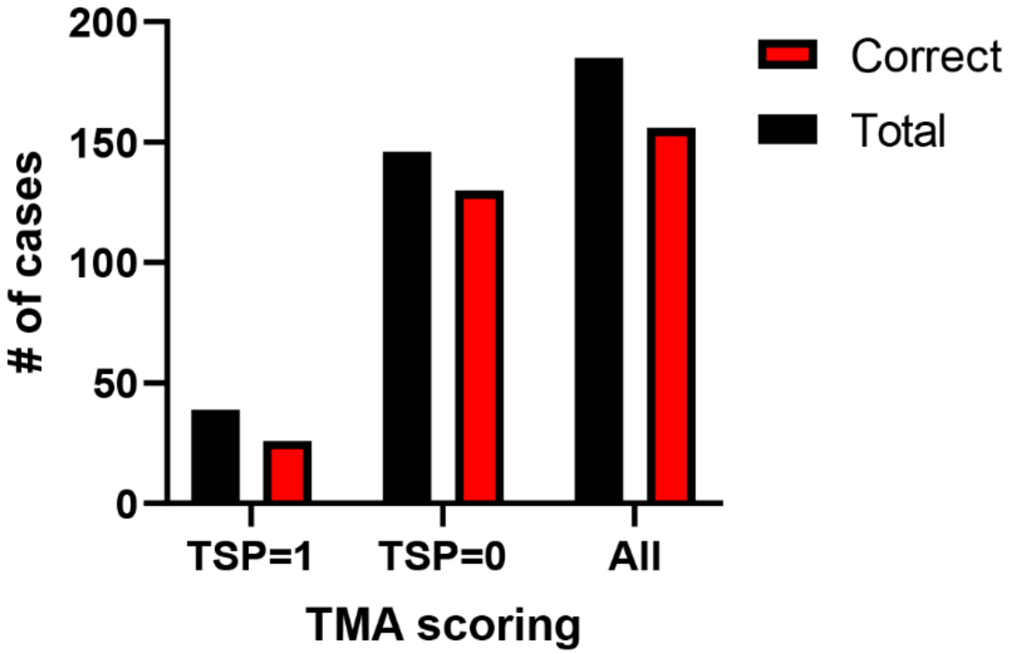

From left to right: high TSP, low TSP, and all cases that were identified in TMAs (black) associated with the respective number of cases that were consistent with TSP assessment in slides (red).

eTable 4. Multivariate Analysis Chemoresistance

Variable	Odds ratio	P-value	[95% conf.	interval]
**TSP**	2.861	**0.01**	1.256	6.515
**Age at diagnosis**	0.991	0.60	0.958	1.025
**Metastases**	2.059	0.10	0.877	4.831
**Residual disease**	1.961	0.10	0.862	4.462
**Lymph Node invasion**	2.580	0.07	0.944	7.049
**Metastases**	1.737	0.23	0.697	4.327

© 2024 Lou E, et al. *JAMA Network Open.*
