# Supplementary material for: Tumor-Stroma Proportion to Predict Chemoresistance in Patients With Ovarian Cancer
Source: JAMA Netw Open. 2024 Feb 27;7(2):e240407. doi: 10.1001/jamanetworkopen.2024.0407 (PMC10900967; doi:10.1001/jamanetworkopen.2024.0407)
Supplement: Supplement 2. — Data Sharing Statement [file jamanetwopen-e240407-s002.pdf]

Data Sharing Statement

Lou. Tumor-Stroma Proportion to Predict Chemoresistance in Patients With Ovarian Cancer. *JAMA Netw Open*. Published February 27, 2024. doi:10.1001/jamanetworkopen.2024.0407

Data
Data available: No
